# Molecular phenotypes of circulating tumor cells and efficacy of nivolumab treatment in patients with head and neck squamous cell carcinoma

**DOI:** 10.1038/s41598-020-78741-0

**Published:** 2020-12-09

**Authors:** Hiroe Tada, Hideyuki Takahashi, Reika Kawabata-Iwakawa, Yurino Nagata, Miho Uchida, Masato Shino, Shota Ida, Ikko Mito, Toshiyuki Matsuyama, Kazuaki Chikamatsu

**Affiliations:** 1 Department of Otolaryngology-Head and Neck Surgery, Gunma University Graduate School of Medicine, 3-39-22, Showa-machi, Maebashi, Gunma 3718511 Japan; 2 Division of Integrated Oncology Research, Gunma University Initiative for Advanced Research, Gunma, 3718511 Japan

**Keywords:** Cancer immunotherapy, Head and neck cancer, Tumour biomarkers

## Abstract

The emergence of immune checkpoint inhibitors (ICIs) has revolutionized the treatment of recurrent/metastatic (R/M) head and neck squamous cell carcinoma (HNSCC). Biomarkers of the therapeutic efficacy of ICIs have been extensively investigated. In this study, we aimed to analyze whether molecular phenotypes of circulating tumor cells (CTCs) are associated with treatment responses and clinical outcomes in patients with R/M HNSCC treated with nivolumab. Peripheral blood samples were collected before treatment initiation and after four infusions of nivolumab. CTCs isolated by depletion of CD45-positive cells were analyzed to determine the expression of *EPCAM*, *MET*, *KRT19*, and *EGFR* using real-time quantitative polymerase chain reaction. CTC-positive samples were analyzed to determine the expression of *PIK3CA*, *CCND1*, *SNAI1*, *VIM*, *ZEB2*, *CD44*, *NANOG*, *ALDH1A1*, *CD47*, *CD274*, and *PDCD1LG2.* Of 30 patients treated with nivolumab, 28 (93.3%) were positive for CTCs. In 20 CTC-positive patients, molecular alterations in CTCs before and after nivolumab treatment were investigated. Patients with *MET*-positive CTCs had significantly shorter overall survival than those with *MET*-negative CTCs (*p* = 0.027). The expression level of *CCND1* in CTCs of disease-controlled patients was significantly higher than that of disease-progressed patients (*p* = 0.034). In disease-controlled patients, the expression level of *CCND1* in CTCs significantly decreased after nivolumab treatment (*p* = 0.043). The *NANOG* expression in CTCs was significantly increased in disease-controlled patients after nivolumab treatment (*p* = 0.036). Our findings suggest that the molecular profiling of CTCs is a promising tool to predict the treatment efficacy of nivolumab.

## Introduction

The immune checkpoint inhibitor (ICI), nivolumab, has shown a significant improvement compared with the investigator’s preferred chemotherapy regimen and is currently used as a new therapeutic option in patients with recurrent and metastatic (R/M) head and neck squamous cell carcinoma (HNSCC)^1–3^. Recently, another ICI, pembrolizumab, has also been reported to have clinical benefit in R/M HNSCC^4^. Thus, the emergence of ICIs has revolutionized in the treatment of R/M HNSCC; however, the objective response rate is still 20%. Therefore, further improvements and identification of biomarkers for therapeutic efficacy are urgently needed.

To date, a variety of candidate biomarkers for R/M HNSCC and other malignancies have been extensively investigated^5–8^. Tumor tissue-derived biomarkers such as programmed death-ligand 1 (PD-L1) expression, interferon-γ signature, CD8 T-cell infiltration, and tumor mutational burden are potential sources for the identification of candidate biomarkers. However, the use of tissue specimens in biomarker discovery for ICIs has raised several concerns, such as tissue quality, tissue heterogeneity, and the time of collection. In particular, the tumor microenvironment might fluctuate with disease progression, treatment pressures, and sites of growth.

Recently, accumulating evidence has indicated that liquid biopsy, including circulating tumor cells (CTCs), cell-free DNA, and exosomes, is a reliable method to obtain real-time tumor information^9–11^. Our previous studies on HNSCC have shown that the presence of CTCs was significantly associated with treatment resistance, locoregional recurrence, and shorter progression-free survival^12^. Notably, the *MET* and *CD274* expression in CTCs showed a poorer and a better prognosis, respectively. Molecular profiling of CTCs may provide new insights to improve patient selection, maximize efficacy, and predict response as one of the more reliable biomarkers. In the present study, we aimed to analyze whether the molecular characteristics of CTCs can be used as biomarkers to predict treatment efficacy in patients with R/M HNSCC treated with nivolumab; we also aimed to investigate the molecular alterations in CTCs during nivolumab treatment.

## Results

### Patient characteristics

A total of 30 patients who had histologically confirmed R/M HNSCC were enrolled in this study. Their characteristics are listed in Table 1. The tumor origins included the paranasal sinus (n = 5), oral cavity (n = 3), nasopharynx (n = 2), oropharynx (n = 5), hypopharynx (n = 12), larynx (n = 2), and parotid gland (n = 1). The treatment responses were as follows: complete response (CR) in 0 (0.0%) patients, partial response (PR) in 6 (20.0%), stable disease (SD) in 6 (20.0%), and progressive disease (PD) in 18 (60.0%) patients. The objective response rate and disease control rate were 20.0% and 40.0%, respectively. Eight patients (26.7%) discontinued 4 courses of nivolumab due to rapid disease progression (n = 6) and intolerable immune-related adverse events (n = 2, intestinal lung disease and colitis), respectively. Although we analyzed clinical features between disease-controlled and disease-progressed patients, there was no significant difference in clinical features evaluated.

**Table 1:** Patient demographics and clinical characteristics.

Clinical variable	Disease-controlled patient	Disease-progressed patient	*P* value
	n = 12	n = 18	
**Age (years): median 67 years old**			
< 67	6	9	> 0.9999
≥ 67	6	9	
**Sex**			
Male	12	14	0.1297
Female	0	4	
**Primary site**			
Paranasal sinus	1	4	0.3884
Oral cavity	2	1	
Nasopharynx	0	2	
Oropharynx	1	4	
Hypopharynx	7	5	
Larynx	1	1	
Parotid gland	0	1	
**Local recurrence**			
(−)	7	8	0.7104
(+)	5	10	
**Lymph node metastasis**			
(−)	7	8	0.7104
(+)	5	10	
**Distant metastasis**			
(−)	5	8	> 0.9999
(+)	7	10	
**Performance status**			
0	10	9	0.1213
1	2	9	
**Previous radiotherapy**			
(−)	3	3	0.6599
(+)	9	15	
**PD-L1 expression**			
< 1%	3	10	0.4197
≥ 1%	6	8	
Unevaluable	3	0	
**Nivolumab treatment**			
< 4 times	3	5	> 0.9999
≥ 4 times	9	13	

Of the 30 patients with R/M HNSCC treated with nivolumab, 28 (93.3%) were positive for CTCs, 20 of whom underwent peripheral blood sample collection at two time points: before treatment initiation and after 4 infusions of nivolumab (Supplementary Fig. 1).

### Molecular phenotype of CTCs and their correlation with treatment responses

**Figure 1:**
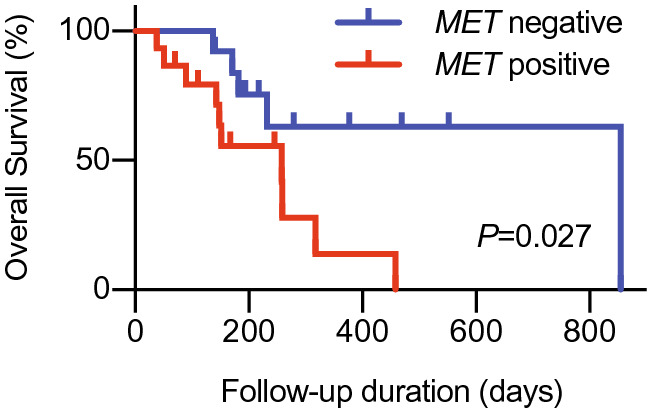
The expression of *MET* in CTCs and correlation with clinical outcome in R/M HNSCC patients treated with nivolumab. The Kaplan–Meier curve and log-rank test for overall survival were performed to compare the differences between patients with *MET*-positive CTCs and those with negative CTCs.

**Figure 2:**
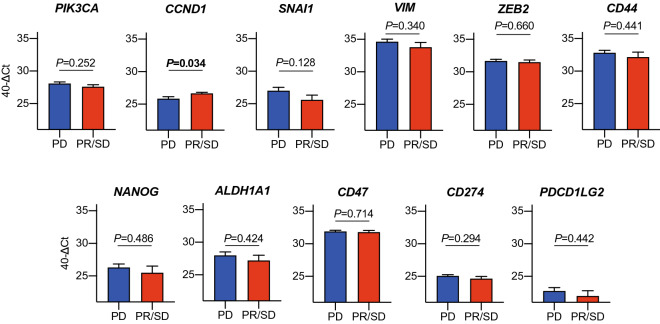
Gene expression in CTCs and treatment responses. The patients with CTCs were divided into two groups according to treatment responses: the disease-controlled group and disease-progressed group. Eleven gene expressions in CTCs were compared.

To investigate the relationship between the molecular phenotype of CTCs and treatment responses, the patients with CTCs were divided into two groups according to treatment responses: disease-controlled and disease-progressed patients. First, we analyzed whether the expression of epithelial-related markers in CTCs before treatment initiation is correlated with treatment efficacy and clinical outcome. There was no significant correlation between the expression of epithelial-related genes in CTCs and treatment efficacy (data not shown); however, patients with *MET*-positive CTCs had a significantly shorter overall survival compared with those with *MET*-negative CTCs (*p* = 0.027) (Fig. 1 and Table 2). We then investigated which gene expression in CTCs was associated with treatment responses. Notably, *CCND1* expression in CTCs from disease-controlled patients was significantly higher than that in disease-progressed patients (*p* = 0.034, Fig. 2). Furthermore, we investigated whether the levels of gene expression in the CTCs was associated with the prognosis; however, there was no relationship between gene expression of CTCs and prognosis in patients treated with nivolumab (Supplementary Table 1).

**Table 2:** Prognostic value of epithelial-related markers in CTCs.

Gene symbol			*P*-value	HR	95% CI
*EPCAM*	+	12	0.3388	1.626	0.5335–4.955
	−	16			
*MET*	+	15	0.0265	3.045	1.082–8.567
	−	13			
*KRT19*	+	25	0.7516	1.265	0.3212–4.980
	−	3			
*EGFR*	+	16	0.9933	1.004	0.3576–2.820
	−	12

### Molecular alterations of CTCs following nivolumab treatment

**Figure 3:**
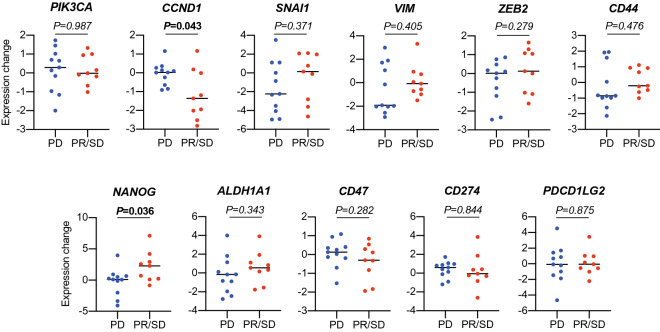
Changes in gene expression in CTCs during nivolumab treatment. The patients with CTCs were divided into two groups according to treatment responses: the disease-controlled group and disease-progressed group. The changes in gene expression in CTCs was compared between the two groups.

We investigated the change in gene expression in CTCs from individual patients during nivolumab treatment. Although there was no significant change in gene expression including PD-L1 in CTCs in all treated patients by nivolumab treatment (data not shown), the expression level of *CCND1* in CTCs in disease-controlled patients, not in disease-progressed patients, was significantly reduced by nivolumab treatment (*p* = 0.043) (Fig. 3). By contrast, the *NANOG* expression levels in CTCs in disease-controlled patients were significantly increased by nivolumab treatment (*p* = 0.036) (Fig. 3).

### TCGA analysis

**Figure 4:**
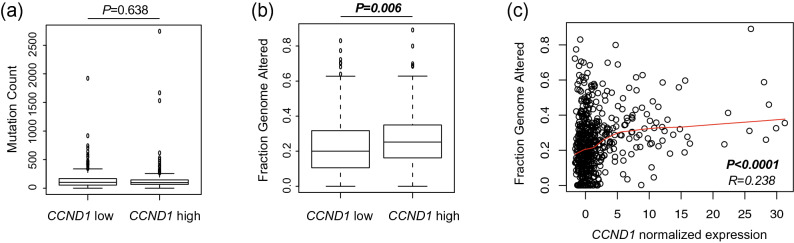
Genomic alterations in head and neck squamous cell carcinoma. Two genomic alterations, tumor mutation counts (**a**) and fraction genome altered (**b**), were compared between tumors with low *CCND1* expression and tumors with high *CCND1* expression. The cutoff value of high and low *CCND1* expression was set as the median. The correlation between the expression of *CCND1* and fraction genome altered (**c**) was evaluated.

To further elucidate the underlying mechanism of *CCND1* that affects the patient’s responses to nivolumab treatment, the relationship between *CCND1* expression and genomic alterations in HNSCC was analyzed using the TCGA database. In 515 patients with HNSCC, although no significant difference was observed between *CCND1* expression and tumor mutation counts, tumors with high *CCND1* expression had significantly higher FGA than those with low *CCND1* expression (*CCND1* expression and tumor mutation counts; *p* = 0.638, *CCND1* expression and FGA; *p* = 0.006, Fig. 4a,b). Moreover, there was a weak correlation between the expression of *CCND1* and FGA (Fig. 4c, r = *0.238*), but not tumor mutation counts (data not shown).

## Discussion

The primary objective of this study was to explore whether the molecular profiling of CTCs could predict treatment efficacy in patients with R/M HNSCC treated with nivolumab. We succeeded in achieving this objective to some extent, and the following points are of particular importance: (1) patients with *MET*-positive CTCs have a poorer prognosis compared with those with *MET*-negative CTCs, (2) CTCs before treatment initiation in the disease-controlled patients showed significantly higher *CCND1* expression and significantly reduced by nivolumab treatment compared with those in disease-progressed patients, and (3) the expression of *NANOG* in CTCs significantly increased in the disease-controlled patients.

The first point probably indicated that *MET*-positive CTCs are resistant not only to chemotherapy and/or radiotherapy but also to immunotherapy. In our previous study on patients with treatment-naïve HNSCC, patients with *MET*-positive CTCs had a shorter progression-free survival than those with *MET*-negative CTCs^12^. c-MET overexpression and amplification in HNSCC have been shown to drive tumorigenesis, proliferation, invasion, and metastasis, and is commonly associated with resistance to therapy^13–16^. With regard to the immunological role of c-Met expression in tumor cells, Saigi et al. demonstrated that c-Met activation induces PD-L1 expression via a pathway independent of JAK/STAT activation^17^. On the contrary, Wang et al. suggested that c-MET expression in tumor cells suppresses the function of NK cells and T cells via the production of indoleamine-2,3-dioxygenase^18^. In addition, the activation of HGF/Met signaling in HNSCC increases glycolysis, which would result in the suppression of T-cell functions^19^. Thus, c-Met expression in tumor cells contributes to immune suppression through a variety of mechanisms. Although the clinical application of ICIs is currently being expanded to a variety of malignancies, a combination of c-Met targeting therapies with ICIs should be considered in patients with *MET*-positive CTCs to improve the therapeutic efficacy of cancer immunotherapy.

The second point suggests that *CCND1* expression in tumor cells might be a good target for T cells reactivated by ICIs. Among several gene expressions related to cell growth, epithelial-mesenchymal transition, cancer stemness, and immune regulation, *CCND1* expression in CTCs is significantly associated with disease control. *CCND1* is known to play a critical role in cell cycle regulation, and high expression of *CCND1* is associated with treatment resistance and poor prognosis in HNSCC, as with *MET*^20–22^. Therefore, the overexpression of cyclin D1 is associated with genome instability, and consequently results in the increase of somatic mutations and neoantigens. In the TCGA database analysis of HNSCC, no significant difference was observed in the in mutation counts between the low *CCDN1* group and high *CCDN1* group; however, the FGA was significantly higher in the high *CCND1* group than in the low *CCND1* group. The FGA is the percentage of genome affected by gains or losses in the copy number, which may increase the neoantigen loads and induce tumor (neoantigen)-specific T cells. Another possibility is that CCND1 protein itself may function as a tumor antigen recognized by T cells. In fact, several epitopes presented by tumor cells and recognized by both CD4 + and CD8 + T cells have been identified^23–26^. Thus, a preexisting immune response against tumor cells expressing *CCND1* may be boosted by nivolumab treatment and thus eliminate such tumor cells. More interestingly, *CCND1* expression in CTCs was significantly decreased in patients with disease control after four infusions of nivolumab, suggesting that tumor-specific T cells may lyse tumor cells expressing *CCND1*.

The last point may indicate that the cancer stemness phenotype represents an immune-resistant phenotype of CTCs. Of patients treated with nivolumab, disease-controlled patients showed significantly increased expression of cancer stemness markers, *NANOG*. This finding suggests that the induction of an effective antitumor immune response by nivolumab treatment presumably allows the concentration of CTCs to display the cancer stem cell (CSC) phenotype due to lysis of non-CSC tumor cells. Although CSCs are known to be subpopulations that are capable of self-renewal and differentiation^27,28^, in terms of immunological characterization of CSCs, CSCs can evade immune surveillance by reducing immunogenicity and immune suppressive activity compared with their non-CSC counterparts^29–32^. Indeed, our previous study revealed that CD44-positive cancer stem-like cells in HNSCC produced various immune suppressive cytokines and enhanced the regulatory T-cell response compared with CD44-negative cells^33^. Hence, development of new treatment strategies targeting CSC-related molecules is needed to further modulate the antitumor immune responses reactivated by ICIs.

Taken collectively, ICIs can boost the immune responses against tumor cells in certain patients and exert a strong selective pressure on tumor cells in peripheral blood as well as within the tumor microenvironment. Despite the small sample size, molecular alterations in CTCs may in fact be a result of the selective pressure of antitumor immune responses induced by nivolumab. Thus, our findings suggest that the molecular profiling of CTCs may be a promising tool to predict the treatment efficacy of nivolumab and provide new insights into more precise immunotherapies.

## Methods

This study was approved by the Gunma University Ethical Review Board for Medical Research Involving Human Subjects (HS2017-152), and all experiments were performed in accordance with the approved guidelines. Written informed consent was obtained from each patient.

### Patients and blood collection

Patients who had histologically confirmed R/M HNSCC and treated with nivolumab were eligible for this study. We evaluated several clinical variables, including age, sex, primary site, local recurrence, lymph node metastasis, distant metastasis, performance status, previous radiotherapy, PD-L1 expression, and completion of 4 course of nivolumab. Treatment response was evaluated according to RECIST, version 1.1, during weeks 8–10. If the patients had an unacceptable level of drug-related toxic effects or rapid disease progression before evaluation, the therapeutic effects were evaluated at that point. Blood samples were collected using the K2EDTA Vacutainer (BD Bioscience) before the first nivolumab infusion and if possible, after fourth nivolumab infusion. The samples were analyzed within 4 h of collection.

### Detection and gene expression analysis of CTCs

CTC detection and gene expression analysis were performed as described previously^34,35^. In brief, the peripheral blood mononuclear cells were isolated from blood samples (7.5 mL), and the contaminating erythrocytes were lysed with a red blood cell lysis buffer (Roche). The cell suspension was incubated with a human CD45 depletion cocktail for 15 min and then with magnetic particles for 10 min (EasySep Human CD45 Depletion Kit II, STEMCELL Technologies). Tubes containing cells were placed on a magnet for 10 min twice, and the unbound cells (CTCs) were transferred to new tubes.

Total RNA from the CTCs was extracted using an RNeasy Micro kit (Qiagen) according to the manufacturer’s instructions. cDNA synthesis was performed using the QuantiTect Reverse Transcription kit (Qiagen) with preamplification using the TaqMan PreAmp Master Mix kit (Applied Biosystems). The preamplified products were then analyzed using real-time quantitative polymerase chain reaction (Applied Biosystems) to quantify the 16 target genes. Sixteen primers for the fifteen targets (epithelial cell adhesion molecule [*EPCAM*]*, MET,* keratin 19 *[KRT19]*, epidermal growth factor receptor [*EGFR*], phosphatidylinositol-4,5-bisphosphate 3-kinase catalytic subunit alpha [*PIK3CA*], cyclin D1 [*CCND1*], snail family transcriptional repressor 1 [*SNAI1*], vimentin [*VIM*], zinc finger E-box binding homeobox 2 [*ZEB2*]*, CD44*, nanog homeobox [*NANOG*], aldehyde dehydrogenase 1 family member A1 [*ALDH1A1*]*, CD47*, *CD274*, and programmed cell death 1 ligand 2 [*PDCD1LG2*]) and *ACTB* (β-actin) as control were purchased from Applied Biosystems (TaqMan Gene Expression Assays). All samples were analyzed in triplicates. Detection of at least one of the four epithelial-related genes (*EPCAM, MET, KRT19,* and *EGFR*) was defined as CTC positivity. The Ct values of the target genes were normalized to a reference gene (*ACTB*). The expression levels and changes of the target genes in CTCs were indicated as 40-delta Ct value and − ΔΔCt value, respectively.

### The Cancer Genome Atlas (TCGA) analysis

We analyzed the relationship between *CCND1* expression and mutation counts and fraction genome altered (FGA) in HNSCC using the TCGA database. The *CCND1* expression Z-scores obtained based on the batch-normalized expression values from Illumina HiSeqV2, mutation count, and FGA values were downloaded from cBioPortal (https://www.cbioportal.org/study/clinicalData?id=hnsc_tcga_pan_can_atlas_2018). The cutoff value of high and low *CCND1* expression was set as the median.

### Statistical analysis

GraphPad Prism version 8.0 for Windows (GraphPad Software, San Diego, CA, USA) was used to perform all statistical analyses. Chi-squared test for independence and Fisher’s exact test were used to examine differences in categorical variables. The Welch’s t-test was used to examine the differences in the means between the two groups. The Kaplan–Meier curves were plotted and compared using the log-rank test. The optimal cut-off values of gene expression in CTCs for overall survival were determined based on receiver operating characteristics curve analysis. In TCGA analysis, the Welch’s t-test was used to determine the significant difference between the high *CCND1* group and low *CCND1* group. The Pearson correlation coefficient was used to measure the strength of a linear association between two variables, *CCND1* expression level and mutation counts or FGA. Two-sided *p*-values < 0.05 were considered significant.

## Supplementary Information

Supplementary Information.

## Abbreviations

ICI: Immune checkpoint inhibitor
HNSCC: Head and neck squamous cell carcinoma
R/M: Recurrent/metastatic
PD-L1: Programmed death-ligand 1
CTC: Circulating tumor cell
TCGA: The Cancer Genome Atlas
FGA: Fraction genome altered
CSC: Cancer stem cell
