# Supplementary material for: Molecular phenotypes of circulating tumor cells and efficacy of nivolumab treatment in patients with head and neck squamous cell carcinoma
Source: Sci Rep. 2020 Dec 9;10:21573. doi: 10.1038/s41598-020-78741-0 (PMC7726556; doi:10.1038/s41598-020-78741-0)
Supplement: Supplementary file 1 — Supplementary Information. [file 41598_2020_78741_MOESM1_ESM.docx]

**Molecular phenotypes of circulating tumor cells and efficacy of nivolumab treatment in patients with head and neck squamous cell carcinoma**

Hiroe Tada^1^, Hideyuki Takahashi^1^, Reika Kawabata-Iwakawa^2^, Yurino Nagata^1^, Miho Uchida^1^, Masato Shino^1^, Shota Ida^1^, Ikko Mito^1^, Toshiyuki Matsuyama^1^, and Kazuaki Chikamatsu^1,^*

^1^Department of Otolaryngology-Head and Neck Surgery, Gunma University Graduate School of Medicine

^2^Division of Integrated Oncology Research, Gunma University Initiative for Advanced Research

***Corresponding author:**

Kazuaki Chikamatsu

Department of Otolaryngology-Head and Neck Surgery, Gunma University Graduate School of Medicine, 3-39-22, Showa-machi, Maebashi, Gunma 3718511, Japan

Phone: +81-27-220-8350

Fax: +81-27-220-8369

Email: tikamatu@gunma-u.ac.jp

**Supplementary Figure 1.** Summary flowchart of this study.


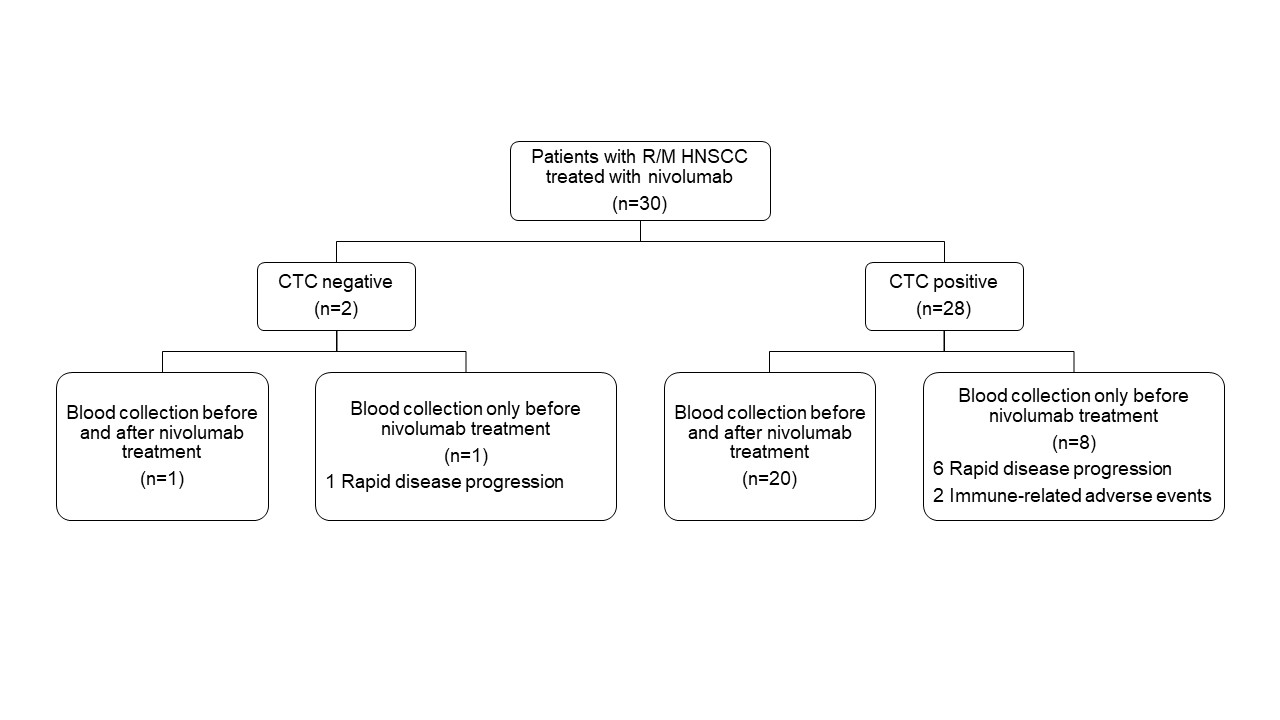


**Supplementary Table 1.** Prognostic value of the gene expression in CTCs.
